# Challenges and Priorities for Pediatric Critical Care Clinician-Researchers in Low- and Middle-Income Countries

**DOI:** 10.3389/fped.2017.00277

**Published:** 2017-12-22

**Authors:** Amelie O. von Saint André-von Arnim, Jonah Attebery, Teresa Bleakly Kortz, Niranjan Kissoon, Elizabeth M. Molyneux, Ndidiamaka L. Musa, Katie R. Nielsen, Ericka L. Fink, The Pediatric Acute Lung Injury and Sepsis Investigators (PALISI) Network

**Affiliations:** ^1^Department of Pediatrics, Division of Pediatric Critical Care, Seattle Children’s Hospital, University of Washington, Seattle, WA, United States; ^2^Department of Global Health, University of Washington, Seattle, WA, United States; ^3^Department of Pediatrics, Division of Critical Care, Washington University, St. Louis, MO, United States; ^4^Department of Pediatrics, Division of Pediatric Critical Care, University of California, San Francisco, San Francisco, CA, United States; ^5^Institute of Global Health Sciences, University of California, San Francisco, San Francisco, CA, United States; ^6^Department of Pediatrics and Emergency Medicine, University of British Columbia and British Columbia Children’s Hospital, Vancouver, Canada; ^7^College of Medicine, University of Malawi, Blantyre, Malawi; ^8^Division of Pediatric Critical Care Medicine, Children’s Hospital of Pittsburgh of UPMC, Pittsburgh, PA, United States

**Keywords:** low- and middle-income countries, low resource settings, researchers, pediatric critical care, support of research, surveys and questionnaires, intensive care unit

## Abstract

**Introduction:**

There is need for more data on critical care outcomes and interventions from low- and middle-income countries (LMIC). Global research collaborations could help improve health-care delivery for critically ill children in LMIC where child mortality rates remain high.

**Materials and methods:**

To inform the role of collaborative research in health-care delivery for critically ill children in LMIC, an anonymous online survey of pediatric critical care (PCC) physicians from LMIC was conducted to assess priorities, major challenges, and potential solutions to PCC research. A convenience sample of 56 clinician-researchers taking care of critically ill children in LMIC was targeted. In addition, the survey was made available on a Latin American PCC website. Descriptive statistics were used for data analysis.

**Results:**

The majority of the 47 survey respondents worked at urban, public teaching hospitals in LMIC. Respondents stated their primary PCC research motivations were to improve clinical care and establish guidelines to standardize care. Top challenges to conducting research were lack of funding, high clinical workload, and limited research support staff. Respondent-proposed solutions to these challenges included increasing research funding options for LMIC, better access to mentors from high-income countries, research training and networks, and higher quality medical record documentation.

**Conclusion:**

LMIC clinician-researchers must be better empowered and resourced to lead and influence the local and global health research agenda for critically ill children. Increased funding options, access to training and mentorship in research methodology, and improved data collection systems for LMIC PCC researchers were recognized as key needs for success.

## Introduction

The United Nations established Millennium Development Goal 4 aimed to reduce the under-five mortality rate by 2/3 between 1990 and 2015 based on the United Nations Millennium Declaration (1). Although overall progress was realized, only 58 of 138 countries achieved these targets with highest rates of childhood deaths concentrated primarily in low- and middle-income countries (LMIC) (2–4). For example, despite a 67% decrease in under 5 mortality rates (U5MR) in Latin America (LA) over the last 25 years, U5MR on average are still 10% higher in LA than in high-income countries (HIC) (5). Investment in resuscitation and critical care improves patient outcomes in LMIC (6–9). However, this health disparity is exacerbated by the “10–90 gap”: only 10% of health-care research expenditures worldwide address diseases that primarily affect the poorest 90% of the world’s population (10).

The high burden of child mortality in LMIC is egregious considering that many lives could be saved by proven, simple resuscitative, and critical care interventions despite austere environments and fewer pediatric critical care (PCC) resources compared with HIC (11–15). There is need for LMIC institutions and researchers to conduct critical care research according to local resources and disease spectra, to disseminate results in-country, and influence policymakers, program managers and medical/public health practice (15, 16). Recent data from LMIC showed very clearly that we cannot translate critical care guidelines from HIC to LMIC, and in fact this practice, especially for sepsis, can be harmful (17–19). As such, the World Health Report 2013 called for LMIC to be not only users, but also producers of health research (16). Such health research requires study of leading regional causes of death and disability, which will provide data necessary to inform allocation, determine health-care delivery strategy, and assess quality standards required for effective critical care (15, 20, 21). Therefore, we conducted a survey with the following objectives: (1) to assess major challenges and potential solutions to PCC research in LMIC; (2) to foster worldwide research collaborations; and (3) to begin building a global research network to promote high quality research focused on improving outcomes for critically ill children.

## Materials and Methods

The Pediatric Acute Lung Injury and Sepsis Investigators (PALISI) Global Health (GH) subgroup (http://www.palisiglobalhealth.org), which includes PCC investigators from Canada and the United States (US), developed an online survey with input from leaders in PCC research in LMIC (Table S1 in Supplementary Material). Survey data were collected and managed using Research Electronic Data Capture (REDCap), an electronic data capture tool, hosted at the University of Washington in Seattle, United States (22). The survey was determined exempt by the Seattle Children’s Institutional Review Board (IRB). The anonymous survey was emailed four times between January and June 2016 to a convenience sample of 56 clinician scientists in LMICs. These clinician scientists were identified by leading PCC researchers from LMIC, and through the PALISI GH subgroup’s network. In addition, survey recipients were asked to forward the survey to LMIC colleagues in the field, and a survey recipient from LA placed this survey on the PCC website “Sociedad Latino Americana de Cuidados Intensivos Pediatricos.” Given that responses were anonymous, we cannot determine between responders from the original convenience sample and responses to the survey posted on the above website. A response rate can therefore not be determined. Descriptive statistics were used for data analysis with subgrouping into respondents’ geographic areas using STATA12.

## Results

### Survey Respondent Characteristics

Forty-seven clinician-researchers from LMIC responded to the survey, the majority from LA (62%) (Table 1). Sixty-six percent of respondents were trained pediatric intensivists, and 23% were intensivists by experience gained through practice. The proportion of trained pediatric intensivists was lowest in Africa (33%). Most respondents worked in combined adult–pediatric intensive care units at urban, public teaching hospitals.

**Table 1:** Survey respondents’ demographics, research background, and involvement.

Survey respondents’ information	Total	SE Asia	Africa	Latin America	Eastern Europe
	*n* = 47 (%)	*n* = 5 (%)	*n* = 12 (%)	*n* = 29 (%)	*n* = 1 (%)
**Credentials**					
Pediatric intensivist	31 (66)	4 (80)	4 (33)	22 (76)	1 (100)
Pediatrician with ICU experience	11 (23)	1 (20)	5 (42)	5 (17)	0 (0)
Other	5 (11)	0 (0)	3 (25)	2 (7)	0 (0)
**ICU beds**					
Less than 7	12 (25)	0 (0)	5 (41)	7 (24)	0 (0)
7–12	16 (34)	3 (60)	4 (33)	8 (28)	1 (100)
More than 12	19 (40)	2 (40)	3 (25)	14 (48)	0 (0)
**Protected research time^a^**					
None	18 (53)	3 (60)	4 (50)	11 (55)	0 (0)
<10 h/week	10 (29)	1 (20)	0 (0)	8 (40)	1 (100)
>10 h/week	5 (15)	1 (20)	3 (38)	1 (5)	0 (0)
**Research training**					
Research degree	5 (15)	0 (0)	0 (0)	4 (20)	1 (100)
University-level coursework	14 (41)	2 (40)	1 (13)	11 (55)	0 (0)
Research seminar	15 (44)	4 (80)	3 (38)	8 (40)	0 (0)
Research project	25 (74)	5 (100)	4 (50)	16 (80)	0 (0)
**Potential research funding sources**					
International collaboration	9 (19)	0 (0)	5 (42)	4 (14)	0 (0)
Local hospital/foundation	19 (40)	3 (60)	2 (17)	14 (48)	0 (0)
Government	2 (4)	1 (20)	1 (8)	0 (0)	0 (0)
International grant	6 (13)	1 (20)	5 (42)	0 (0)	0 (0)
None	18 (38)	2 (40)	3 (25)	12 (41)	1 (100)
Currently funded research	7 (21)	1 (20)	3 (40)	3 (15)	0 (0)
Current research involvement	34 (72)	5 (100)	8 (67)	20 (69)	1 (100)
Clinical observational	27 (79)	5 (100)	6 (75)	15 (75)	1 (100)
Randomized controlled trial	14 (41)	2 (40)	3 (38)	9 (45)	0 (0)
Quality improvement	15 (44)	3 (60)	4 (50)	8 (40)	0 (0)
Research publication	18 (53)	3 (60)	5 (63)	10 (50)	0 (0)
**Research role**					
Principal investigator	20 (59)	4 (80)	6 (75)	9 (45)	1 (100)
Coinvestigator	10 (29)	1 (20)	2 (25)	7 (35)	0 (0)
Data collector	2 (6)	0 (0)	0 (0)	2 (10)	0 (0)
Local research network	12 (26)	2 (40)	4 (33)	6 (21)	0 (0)

*SE, South East; ICU, intensive care unit*.

*^a^Nine respondents were not actively involved in a research project at the time of survey participation and did not respond to this question*.

### Respondents’ Research Involvement and Resources

Active research involvement was reported by 34 (72%) respondents, all of whom had completed variable amounts of training in research methodology (Table 1). Fifty-eight percent were principal investigators (PIs) and 29% Co-PIs. Most respondents classified their research focus as clinical (82%), as compared with basic science (2%) and quality improvement (QI) (3%). Forty-seven percent had protected research time, and of those, 29% reported no more than 10 h of dedicated research time per week (Table 1). Twenty-one percent of active researchers, mostly from Africa, had research funding, and 53% reported publishing their results in a peer-reviewed journal.

### Current Research Priorities, Challenges, and Potential Solutions

Most respondents considered research of high importance (Table 2). Generating data that improved clinical care (51%) and establishing guidelines to standardize care (28%) were the most important reasons to perform research involving critically ill children. The main personal benefits cited were opportunities for research collaboration (70%), peer recognition (62%), and career advancement (40%).

**Table 2:** Importance of challenges and solutions to pediatric critical care (PCC) research in LMIC.

Research description, challenges and solutions	Total	SE Asia	Africa	Latin America	Eastern Europe
	*n* = 47 (%)	*n* = 5 (%)	*n* = 12 (%)	*n* = 29 (%)	*n* = 1 (%)
**Importance of PCC research in LMIC**					
Critical	18 (38)	3 (60)	5 (42)	9 (31)	1 (100)
Very important	20 (43)	1 (20)	4 (33)	15 (52)	0 (0)
Important	9 (19)	1 (20)	3 (25)	5 (17)	0 (0)
**Most important reason to do research**					
Affect positive change in clinical care	24 (51)	3 (60)	6 (50)	14 (48)	1 (100)
Establish guidelines/protocols	13 (28)	2 (40)	3 (25)	8 (28)	0 (0)
Greater understanding of disease	2 (4)	0 (0)	0 (0)	1 (3)	0 (0)
Increase resource availability	3 (6)	0 (0)	2 (17)	1 (3)	0 (0)
Quality and safety	4 (9)	0 (0)	1 (8)	3 (10)	0 (0)
**Key areas of research in LMIC**					
Sepsis	18 (38)	2 (40)	3 (25)	12 (41)	1 (100)
Trauma	12 (26)	0 (0)	1 (8)	10 (34)	1 (100)
Invasive and non-invasive positive pressure ventilation	14 (30)	0 (0)	3 (25)	11 (38)	0 (0)
Quality improvement and patient safety	11 (23)	2 (40)	2 (17)	7 (24)	0 (0)
Health-care associated infections	9 (19)	3 (60)	2 (17)	4 (14)	0 (0)
ARDS/ALI	7 (15)	1 (20)	0 (0)	6 (21)	0 (0)
Cost-effectiveness	7 (15)	1 (20)	5 (42)	1 (3)	0 (0)
Education/capacity building	6 (13)	1 (20)	2 (17)	3 (10)	0 (0)
Nutrition	6 (13)	2 (40)	0 (0)	4 (14)	0 (0)
Mortality risk/severity of illness scores	5 (11)	1 (20)	0 (0)	4 (14)	0 (0)
Ethics and palliative care	5 (11)	0 (0)	1 (8)	4 (14)	0 (0)
**Personal benefits of research**					
Research collaboration	33 (70)	3 (60)	6 (50)	23 (79)	1 (100)
Peer recognition	29 (62)	3 (60)	7 (58)	18 (62)	1 (100)
Career advancement	19 (40)	3 (60)	6 (50)	9 (31)	1 (100)
Salary	6 (13)	0 (0)	4 (44)	2 (7)	0 (0)
Other	4 (9)	2 (40)	1 (8)	1 (3)	0 (0)
**Research challenges**					
Lack of funding	28 (60)	4 (80)	8 (67)	16 (55)	0 (0)
High clinical burden	22 (47)	3 (60)	3 (25)	15 (52)	1 (100)
Limited research support staff	22 (47)	3 (60)	3 (25)	16 (55)	0 (0)
IRB issues	12 (26)	0 (0)	4 (33)	7 (24)	1 (100)
Lack of statistical support	12 (26)	2 (40)	0 (0)	9 (31)	1 (100)
Limited research training opportunities	10 (21)	1 (20)	3 (25)	6 (21)	0 (0)
Finding research mentor(s)	7 (15)	0 (0)	5 (42)	2 (7)	0 (0)
Difficulty with publications	8 (17)	1 (20)	2 (17)	5 (17)	0 (0)
Lack of reliable medical records	4 (9)	0 (0)	3 (25)	1 (3)	0 (0)
Study subject recruitment	4 (9)	0 (0)	1 (8)	3 (10)	0 (0)
**Research solutions**					
More funding for low resource settings	33 (70)	5 (100)	8 (67)	19 (66)	1 (100)
Access to mentors in HIC	24 (51)	3 (60)	8 (67)	13 (45)	0 (0)
Access to research training	21 (45)	0 (0)	4 (33)	16 (55)	1 (100)
Access to research network	18 (38)	2 (40)	3 (25)	13 (45)	0 (0)
Improved medical records	16 (34)	2 (40)	3 (25)	10 (34)	1 (100)
Opportunities to present	9 (19)	1 (20)	3 (25)	5 (17)	0 (0)
Streamlined IRB review	6 (13)	0 (0)	3 (25)	3 (10)	0 (0)
Protected research time	4 (9)	2 (40)	0 (0)	2 (7)	0 (0)
HIC research collaborations	27 (57)	4 (80)	8 (67)	14 (48)	1 (100)
**Importance of collaboration**					
Critical	4 (9)	0 (0)	3 (25)	0 (0)	1 (100)
Very	12 (26)	3 (60)	3 (25)	6 (21)	0 (0)
Important	6 (13)	0 (0)	1 (8)	5 (17)	0 (0)
Less	3 (6)	1 (20)	0 (0)	2 (7)	0 (0)
Not	1 (2)	0 (0)	0 (0)	1 (3)	0 (0)
**Benefits of collaboration**					
Research training	21 (45)	0 (0)	6 (50)	14 (48)	1 (100)
Experience in manuscript preparation	19 (40)	2 (40)	4 (33)	12 (41)	1 (100)
Established guidelines	16 (34)	3 (60)	3 (25)	9 (31)	1 (100)
Financial support	13 (28)	0 (0)	5 (42)	8 (28)	0 (0)
Medical supplies	9 (19)	0 (0)	6 (50)	3 (10)	0 (0)
Other	5 (11)	1 (20)	2 (17)	2 (7)	0 (0)
**Problems with collaboration**					
Lack of understanding of local setting	18 (38)	1 (20)	6 (50)	11 (38)	0 (0)
Lack of sustainable benefit	12 (26)	0 (0)	6 (50)	6 (21)	0 (0)
Communication limitations	12 (26)	0 (0)	3 (25)	1 (3)	0 (0)
Lack of data sharing	9 (19)	1 (20)	1 (8)	6 (21)	1 (100)
Lack of local research administration	7 (15)	2 (40)	3 (25)	2 (7)	0 (0)
Lack of proper acknowledgment	7 (15)	1 (20)	1 (8)	4 (14)	1 (100)
Dissimilar vision and goals	7 (15)	0 (0)	1 (8)	6 (21)	0 (0)
Ethical conflict	4 (9)	1 (20)	0 (0)	3 (10)	0 (0)
Political barriers	4 (9)	0 (0)	3 (25)	1 (3)	0 (0)
Lack of high tech equipment	1 (2)	0 (0)	1 (8)	0 (0)	0 (0)
Benefits of collaboration outweigh problems	29 (62)	4 (80)	8 (67)	16 (55)	1 (100)

*LMIC, low- and middle-income counties; HIC, high-income countries; SE, South East; ARDS/ALI, acute respiratory distress syndrome/acute lung injury; IRB, Institutional Review Board*.

Sepsis was the most common research area (38%), followed by invasive/non-invasive mechanical ventilation management (30%), trauma/traumatic brain injury (26%), and QI/patient safety (23%). Key research topics varied by region, with African respondents emphasizing cost-effectiveness, while South East (SE) Asian and LA respondents favored sepsis.

The highest-rated challenges to performing PCC research in LMIC were lack of funding (60%), few research support staff (47%), and high clinical workload (47%). A higher proportion of African researchers found it challenging to find mentors (42%); whereas LA researchers lacked statistical support (31%). The highest-rated solutions to these challenges were increasing research funding options for LMIC (70%), better access to mentors from HIC (51%), improved access to research training (45%), and better medical record keeping (34%).

Research networks were available to 26% of responders, to which LA researchers had the least access. Over half of the respondents collaborated with HIC researchers. These relationships were critically important for 9%, very important or important for 26 and 13% of LMIC clinician-researchers, respectively. The benefits of HIC collaborations included obtaining formal research training and experience (45%); gaining experience in manuscript preparation and publication (40%); and using established guidelines, protocols, and pathways to guide protocol development (34%). The top-rated benefits varied slightly by geographic region (Table 2). The primary challenges of HIC research collaboration were a lack of understanding of local settings (38%), communication limitations (26%), and the inability to sustain benefits over time (26%). Overall, 62% of researchers in LMICs thought the benefits of collaboration outweighed the difficulties.

## Discussion

This study provides information on the vast needs and enormous challenges facing PCC clinician-researchers in LMIC. Most respondents rated the importance of research in PCC to improving health outcomes very highly, but cited lack of funding, heavy clinical workload, and limited research staff as major disincentives. Highest-rated solutions included increased funding opportunities and access to HIC mentors and research training.

Currently, no LMIC invests >1% of its gross domestic product on research and development, as compared with 2–4% in Korea, Japan, the US, and the United Kingdom (17, 23). HIC funders may insist that principal recipients of grants are based in donor countries, thus excluding LMIC institutions from receiving overhead support. It is not surprising then that only 21% of respondents were able to obtain funding and 60% lacked the financial support to conduct research. Lack of trained personnel is also a barrier; however, some solutions exist, such as the US National Institute of Health Fogarty Emerging Global Leader Award program, the NHLBI-UnitedHealth Global Health Centers of Excellence Program, and the World Health Organization/Special Programme for Research and Training in Tropical Diseases Career Development Fellowship, which support scientists from LMIC (24). These programs are promising and are partly facilitated in LMIC, but successful applicants need established mentorship and if locally not available, mentors will come from HIC. Global networking should not be daunting because frugal technology is ubiquitous, hence building research communities, and linking like-minded scientists is relatively easy and highly beneficial. Improving record keeping was also rated as one of the main solutions to research challenges. In low-income settings, there is considerable pressure to contain the cost of data acquisition and still implement effective data management frameworks that produce quality data while research staff and physician time are limited. Introduction of the data capture tool REDCap, a non-commercial software solution designed for rapid development and deployment of electronic data, to a multi-site clinical information and research network in Kenya is now delivering quality pediatric data for clinical improvement and research use (25). This low-cost, sustainable and scalable program could be a solution for other resource-limited settings.

Physician-investigators in HIC face similar challenges as faculty in LMIC, which include securing funding for research programs; promotion or tenure systems not being responsive to the different needs of faculty working in both research and clinical care; and the increasing burdens of clinical care (26, 27). Problems with workload and burnout have been described for intensivists in HIC (28). Equivalent literature is lacking from LMIC, but given that most of sub-Saharan Africa has less than 0.5 doctors, and a large portion of LA less than 1.5 doctors per 1,000 population, compared with 2.5 and 3–4 per 1,000 in North America and Western Europe, respectively (29), clinician-researchers in LMIC are likely more stretched than in HIC. The contrast for numbers of PCC providers between LMIC and HIC is even more stark and likely plays into a PCC clinician-researchers workload dilemma: in LA the number of PCC physicians ranges between 2 in Honduras and 318 in Mexico, respectively; Kenya has a total of 3 PCC physicians serving more than 21 million people less than 18 years of age; compared with 1,805 PCC doctors in the US for 78 million children and adolescents (30–32).

The vast majority of LMIC researchers involved in collaborations with HIC rated these relationships as important for their research, emphasizing the value of nurturing and sustaining these partnerships. The main benefit of collaborations was the opportunity to be trained and gain experience in research, protocol, and guideline development. Mentoring was important, as well as the need for equal, mutually beneficial partnerships. Recent qualitative studies from LMIC highlight common themes of poorly distributed benefits from research involvement, and poor translation of research into local settings (21, 23).

Researchers in LMIC should have opportunities to obtain research funding to provide protected time for research. Research partnerships are often unequal; LMIC researchers should be included in research protocol development, grant applications, and investigation leadership. Funding often depends on successful research track records; if LMIC researchers are not given the opportunity to be PIs or gain acknowledgment for their work, they will continue to be disenfranchised. Mentorship should be complementary of expertise, experience, and understanding, and foster symbiotic relationships. Previous high-impact studies have proven that HIC–LMIC collaboration can successfully improve clinical care, directly benefit the participating communities and give career opportunities to local researchers (17, 33, 34). This should be the paradigm for PCC research study design in LMIC.

Association with experienced PALISI researchers and mentorship in grant writing could help address the funding problems of LMIC PCC researchers. To help address barriers to research in LMIC identified in this survey, the PALISI GH subgroup has begun outreach to PCC researchers in LMIC. Specifically, some PCC researchers have presented research proposals (in-person and *via* webinar) for purposes of feedback and/or recruitment of sites, while others have identified mentors and collaborators. The PALISI-GH subgroup supports the development of PALISI-like groups in LMIC to increase growth of research collaborations and research investigator development. PALISI provides annual research training opportunities for North American PCC fellows, which could be modeled by LMIC countries or potentially be offered to LMIC researchers *via* remote participation or subsidized for in-person attendance by grants.

Although this survey generated interesting data regarding PCC research in LMIC, it had several limitations. The low total number of survey responses limits the generalizability of the results, especially for SE Asia and Eastern Europe. Responses from LA were higher likely due to better local survey publicizing. A response rate cannot be determined given that respondents *via* original invitation versus website survey cannot be distinguished. Given some of the survey recipients were acquired through the PALISI GH subgroup’s network, responses may be biased. The term “local research network” was not specifically defined in the survey and could have been interpreted differently by responders, such as hospital, regional, country or continent-wide networks.

## Conclusion

Since over 95% of the global under-five mortality still occurs in LMIC, researchers from these regions must play a role in setting research priorities, developing clinical guidelines for their settings, informing national policy and improving care for their communities. Increased funding options; access to training and mentorship in research methodology, to research networks, and improved data collection systems are paramount for the success of LMIC PCC researchers. Even in the absence of independent funding mechanisms, HIC–LMIC collaborations as well as regional and international research networks such as PALISI GH can provide important support to colleagues around the world.

## Ethics Statement

This study was carried out in accordance with the recommendations of the Seattle Children’s Institutional Review Board, which determined the study to be exempt from IRB approval, since the human subject research involved the use of survey procedures; and the survey did not record information in such a manner that human subjects could be identified, directly or through identifiers linked to the subjects; and any disclosure of the human subjects’ responses outside the research could not reasonably place the subjects at risk of criminal or civil liability or be damaging to the subjects’ financial standing, employability, or reputation.

## Author Contributions

AA was involved in survey design, obtained IRB approval, distributed the survey, and participated in data analysis and manuscript writing. EF was involved in survey design, assisted in survey distribution, and participated in data interpretation and in manuscript writing. JA, NK, and EM were involved in survey design, data interpretation, and manuscript writing. NM was involved in manuscript writing. KN completed the data analysis and was involved data interpretation and manuscript writing. TK was involved in data interpretation and manuscript writing.

## Conflict of Interest Statement

The authors declare that the research was conducted in the absence of any commercial or financial relationships that could be construed as a potential conflict of interest.
