# Supplementary material for: Challenges and Priorities for Pediatric Critical Care Clinician-Researchers in Low- and Middle-Income Countries
Source: Front Pediatr. 2017 Dec 22;5:277. doi: 10.3389/fped.2017.00277 (PMC5744187; doi:10.3389/fped.2017.00277)
Supplement: Supplementary file 1 [file table_1.PDF]

Survey of Pediatric Critical Care Clinician-Researchers in Low to Middle Income Countries

Dear Colleague,

We invite you to participate in this brief survey on the state of pediatric critical care research activities in limited-resource settings.

This survey was developed by the Pediatric Acute Lung Injury & Sepsis Investigators (PALISI) Global Health subgroup.

The objectives of this survey are to report on the state of research activities in the field of pediatric critical illness in Low, Lower-Middle and Middle Income countries, address current barriers to research in this field and setting, prioritize research needs, and promote research networks and collaborations.

Survey participation is voluntary. Participation is anonymous, unless you choose to leave your contact information at the end of the survey. The Seattle Children's institutional review board (IRB) has determined this survey exempt from IRB review.

The survey will take 5-10 minutes to complete. Thank you for your interest and time in responding to this survey.

Please forward this survey to your colleagues from Low, Lower-Middle and Middle Income countries involved in pediatric critical care research.

Sincerely,

Amelie von Saint Andre, Ericka Fink, Niranjan (Tex) Kissoon

and the PALISI Global Health subgroup

For questions, please contact ameliev@uw.edu or FinkEL@ccm.upmc.edu.

Respondent Demographics

What city and country do you live in? __________________________________

What are your professional credentials?
- ◯ Pediatric intensivist by training
- ◯ Pediatrician with ICU experience
- ◯ General practitioner with PICU experience
- ◯ Nurse/nurse practitioner
- ◯ Other, please specify (write-in)

Other credentials __________________________________

For how many years have you practiced critical care medicine? __________________________________

Are you involved in research?
- ◯ Yes
- ◯ No

How much protected time do you have for research?
- ◯ Full time research
- ◯ > 10 hours / week
- ◯ 1-10 hours / week
- ◯ None

What is your primary research category?
- ◯ Basic science
- ◯ Clinical
- ◯ Quality improvement
- ◯ Other, please specify:

Other research category ______________________________

What is your primary research subject?

____

What is your research experience and training? (Please check all that apply)
- ☐ Advanced degree in research methodology (i.e. Masters, PhD)
- ☐ University-level courses in research methodology
- ☐ Research methodology seminar at conference (local, national or international)
- ☐ Participation in research project
- ☐ Other, please specify:
- ☐ None

Other experience ______________________________

What resources do you have available in the area where you care for the critically-ill or in your ICU?

Basic Resources

	Not available	Intermittently available	Reliably available
Clean Water	◯	◯	◯
Electricity	◯	◯	◯

Respiratory Support

	Not available	Intermittently available	Reliably Available
Nasal cannula oxygen	◯	◯	◯
Face mask oxygen	◯	◯	◯
Non-rebreather oxygen	◯	◯	◯
CPAP	◯	◯	◯
Non-invasive mechanical ventilation	◯	◯	◯
Invasive mechanical ventilation	◯	◯	◯

Extracorporeal support

	Not available	Intermittently available	Reliably Available
Dialysis capability (either hemodialysis or peritoneal dialysis)	○	○	○
Extracorporeal membrane oxygenation capability (ECMO)	○	○	○

Monitoring

	Not available	Intermittently available	Reliably available
Intracranial pressure monitoring	○	○	○
Electroencephalography	○	○	○

Blood Bank

	Not available	Intermittently available	Reliably available
Blood bank for transfusion	○	○	○

Maintenance

	Not available	Intermittently available	Reliably available
Maintenance personnel for medical equipment (i.e., mechanical ventilators)	○	○	○

Laboratory services

	Not available	Intermittently available	Reliably available
Microbiological cultures (i.e., blood, cerebrospinal fluid)	○	○	○
Electrolytes	○	○	○
Blood counts	○	○	○
Kidney function	○	○	○
Liver function	○	○	○
Blood gas analysis	○	○	○

Echocardiogram

	Not available	Intermittently available	Reliably available
Echocardiogram	○	○	○

Transport

	Not available	Intermittently available	Reliably available
Air transport	○	○	○
Ground transport	○	○	○

Other characteristics of your hospital

What patient age range does your ICU or area for critically-ill patients serve? ______________________________

What best describes your hospital's characteristics? Please check all that apply.

- ☐ Rural
- ☐ Suburban
- ☐ Urban
- ☐ Private
- ☐ Public
- ☐ Teaching hospital
- ☐ Non-teaching hospital
- ☐ Free-standing children's hospital

How many pediatric beds does your hospital have? ______________________________

How many pediatric hospital admissions occur per year? ______________________________

How many beds does your ICU have? ______________________________

How many ICU admissions occur per year? ______________________________

How many doctors total are available to work in the ICU? ______________________________

How many doctors work in the ICU during the day? ______________________________

How many trainee doctors work in the ICU during the day? ______________________________

How many trainee doctors total are available to work in the ICU? ______________________________

How many nurses total are available to work in the ICU? ______________________________

How many respiratory therapists total are available to work in the ICU? ______________________________

How many respiratory therapists work in the ICU during the day? ______________________________

What is the average bedside nurse:patient ratio in your ICU during the day?

- ◯ 1:1
- ◯ 1:2
- ◯ 1:3
- ◯ 1:4
- ◯ 1:5
- ◯ > 1:5

What are the top 5 diagnoses for admission to your ICU?

____

Research

How important do you think conducting research in your community is for improving your patient's health?

- ◯ Not important
- ◯ Less important
- ◯ Important
- ◯ Very important
- ◯ Critically important

What would be your most important reasons to perform research involving critically-ill children?

- ◯ To generate data that affects positive change in clinical care
- ◯ To establish guidelines/protocols/pathways to standardize care
- ◯ To gain greater understanding of specific diseases
- ◯ To establish quality and safety assurance
- ◯ To increase availability of resources (i.e., medical supplies, equipment)
- ◯ Other, please describe:

Other

____

What are the 5 key areas of research that you feel are crucial to study for pediatric critical care in resource-poor settings?

____

What types of research are you currently participating in that involve critically-ill children ? Check all that apply.

- ☐ Clinical Research: Observational
- ☐ Clinical Research: Randomized Control Trial
- ☐ Clinical Research: Case/Case Series
- ☐ Quality Improvement/Quality Assurance Research
- ☐ Basic Science Research

For each project, please describe:

Your role (Primary investigator, co-investigator, data collection, etc.)

____

The research topic or question

Was your research funded?

- ◯ Yes
- ◯ No

If yes, please list source (government grant, foundation, etc.)

____

Was this published in a peer reviewed journal?

- ◯ Yes
- ◯ No

If yes, please upload publication

What are the major challenges of performing research in your settings? Please rank the top 3 major challenges.

Research Challenge #1

- ◯ No ethical review board available
- ◯ Administrative time burden to get ethical review board approval
- ◯ Ethical review board requirements too burdensome
- ◯ Cost to obtain ethical review board approval
- ◯ Cultural issues with consenting patients
- ◯ Lack of research funding sources
- ◯ Limited pool of trained research support staff
- ◯ Lack of availability of reliable medical records
- ◯ Lack of statistical support, data management personnel
- ◯ Difficulty with publication of research data
- ◯ Finding research mentor(s)
- ◯ Limited opportunities for research training
- ◯ Recruitment of study subjects
- ◯ Clinical burden too large - no time for research
- ◯ Other, please describe: (free text)

Other research challenge ________________________________

Research Challenge #2

- ◯ No ethical review board available
- ◯ Administrative time burden to get ethical review board approval
- ◯ Ethical review board requirements too burdensome
- ◯ Cost to obtain ethical review board approval
- ◯ Cultural issues with consenting patients
- ◯ Lack of research funding sources
- ◯ Limited pool of trained research support staff
- ◯ Lack of availability of reliable medical records
- ◯ Lack of statistical support, data management personnel
- ◯ Difficulty with publication of research data
- ◯ Finding research mentor(s)
- ◯ Limited opportunities for research training
- ◯ Recruitment of study subjects
- ◯ Clinical burden too large - no time for research
- ◯ Other, please describe: (free text)

Other research challenge ________________________________

Research Challenge #3

- ◯ No ethical review board available
- ◯ Administrative time burden to get ethical review board approval
- ◯ Ethical review board requirements too burdensome
- ◯ Cost to obtain ethical review board approval
- ◯ Cultural issues with consenting patients
- ◯ Lack of research funding sources
- ◯ Limited pool of trained research support staff
- ◯ Lack of availability of reliable medical records
- ◯ Lack of statistical support, data management personnel
- ◯ Difficulty with publication of research data
- ◯ Finding research mentor(s)
- ◯ Limited opportunities for research training
- ◯ Recruitment of study subjects
- ◯ Clinical burden too large - no time for research
- ◯ Other, please describe: (free text)

Other research challenge ________________________________

Rank the potential solutions to these challenges. Please rank the top 3.

Solution #1
- ◯ Improved medical record keeping
- ◯ Streamlined ethics review
- ◯ Research network with centralized resource lists
- ◯ More funding designated for low resource settings
- ◯ Access to research training
- ◯ Opportunities to present research
- ◯ Access to mentors in high resourced countries
- ◯ Other

Other solution ______________________________

Solution #2
- ◯ Improved medical record keeping
- ◯ Streamlined ethics review
- ◯ Research network with centralized resource lists
- ◯ More funding designated for low resource settings
- ◯ Access to research training
- ◯ Opportunities to present research
- ◯ Access to mentors in high resourced countries
- ◯ Other

Other solution ______________________________

Solution #3
- ◯ Improved medical record keeping
- ◯ Streamlined ethics review
- ◯ Research network with centralized resource lists
- ◯ More funding designated for low resource settings
- ◯ Access to research training
- ◯ Opportunities to present research
- ◯ Access to mentors in high resourced countries
- ◯ Other

Other solution ______________________________

Have you collaborated with researchers in high income countries?
- ◯ Yes
- ◯ No

How important is this for your research?
- ◯ Not important
- ◯ Less important
- ◯ Important
- ◯ Very important
- ◯ Critically important

What country(ies) were your collaborators from? ______________________________

What were some benefits of this collaboration? Click all that apply.
- ☐ Formal research training and experience
- ☐ Availability of medical supplies
- ☐ Experience in manuscript preparation/publication
- ☐ Established guidelines/protocols/pathways to guide protocol development
- ☐ Financial support
- ☐ Other, please describe

Other benefits ______________________________

What were some problems experienced in collaborating with researchers from high-income countries? Please rank the top 3.

Problem #1

- ○ Inability to sustain benefit
- ○ Ethical conflict(s)
- ○ Communication limitations
- ○ Political barriers
- ○ Dissimilar vision(s) goals
- ○ Lack of understanding of local setting
- ○ Lack of local research administration
- ○ Lack of proper acknowledgement in publications/presentations
- ○ Lack of access to data-sharing
- ○ Other

Other problem __________________________________

Problem #2

- ○ Inability to sustain benefit
- ○ Ethical conflict(s)
- ○ Communication limitations
- ○ Political barriers
- ○ Dissimilar vision(s) goals
- ○ Lack of understanding of local setting
- ○ Lack of local research administration
- ○ Lack of proper acknowledgement in publications/presentations
- ○ Lack of access to data-sharing
- ○ Other

Other problem __________________________________

Problem #3

- ○ Inability to sustain benefit
- ○ Ethical conflict(s)
- ○ Communication limitations
- ○ Political barriers
- ○ Dissimilar vision(s) goals
- ○ Lack of understanding of local setting
- ○ Lack of local research administration
- ○ Lack of proper acknowledgement in publications/presentations
- ○ Lack of access to data-sharing
- ○ Other

Other problem __________________________________

Do the benefits of collaboration outweigh the problems?

- ○ Yes
- ○ No

What are the major benefits to you personally for performing research in your setting (please select all that apply)?

- ☐ Increased salary benefits
- ☐ Peer recognition of expertise
- ☐ Opportunities for research collaboration
- ☐ Career advancement (promotion)
- ☐ Other, please describe

Other benefits __________________________________

What is the typical source of funding for research in your region (select all that apply)?

- ☐ None usually available
- ☐ Collaborating with international investigators
- ☐ Local foundation/hospital
- ☐ Government
- ☐ Applying for international grants

Is there a local network of researchers with whom you have collaborated with or know about?

- ○ Yes
- ○ No

Please provide network name and contact information if available. ____

Please provide your contact information for future collaboration and research network membership. Please note that providing your contact information is voluntary. ____
